# SGC-CK2-1 Is an Efficient Inducer of Insulin Production and Secretion in Pancreatic β-Cells

**DOI:** 10.3390/pharmaceutics14010019

**Published:** 2021-12-22

**Authors:** Mandy Pack, Claudia Götz, Selina Wrublewsky, Mathias Montenarh

**Affiliations:** 1 Medical Biochemistry and Molecular Biology, Saarland University, Building 44, D-66421 Homburg, Germany; mandy.pack@web.de (M.P.); mathias.montenarh@uks.eu (M.M.); 2 Institute for Clinical & Experimental Surgery, Saarland University, D-66421 Homburg, Germany; selina.wrublewsky@uks.eu

**Keywords:** protein kinase CK2, SGC-CK2-1, CX-4945, pancreatic β-cells, insulin

## Abstract

The pyrazolopyrimidine based compound SGC-CK2-1 is a potent and highly specific CK2 inhibitor and a new tool to study the biological functions of protein kinase CK2 irrespective from off-target effects. We used this compound in comparison with the well-established CK2 inhibitor CX-4945 to analyze the importance of CK2 for insulin production and secretion from pancreatic β-cells. Both inhibitors affected the proliferation and viability of MIN6 cells only marginally and downregulated the endogenous CK2 activity to a similar level. Furthermore, both inhibitors increased the message for insulin and boosted the secretion of insulin from storage vesicles. Thus, regarding the high specificity of SGC-CK2-1, we can clearly attribute the observed effects to biological functions of protein kinase CK2.

## 1. Introduction

The human kinome encompasses more than 500 kinases [1]. One of these kinases, namely CK2 (formerly known as casein kinase 2) phosphorylates a great number of proteins which are implicated in central biological processes leading to the difference between life and death of a cell, cell proliferation, DNA damage response, differentiation of cells, controlling of angiogenesis, and ion channel activation [2,3,4,5,6,7]. In addition, metabolic pathways including the glucose metabolism are regulated by CK2 [8]. CK2 phosphorylates and controls a number of different transcription factors that regulate the expression of various genes. Over the last 20 years, it has been shown that the kinase activity of CK2 is elevated in rapidly proliferating cells, such as tumor cells (for review see: [9,10]). Furthermore, it was shown that CK2 is a potent suppressor of apoptosis, which is a hallmark of cancer [11]. Due to this particular property of CK2, there are many attempts to inhibit the kinase activity in order to stop cell proliferation and tumor growth [10,12,13]. Meanwhile, there is a still growing list of CK2 inhibitors, most of which are ATP competitive inhibitors [14]

It has been shown that the CK2 kinase inhibitors DRB and DMAT lowered glucose-induced insulin secretion [15]. In contrast to these results, over the last more than ten years, we and others [16] have shown that CK2 is strongly implicated in the regulation of the expression of insulin in pancreatic β-cells. We have demonstrated that inhibition of CK2 led to an increased transcription of the gene encoding insulin in pancreatic β-cells [17]. The elevated level of insulin production is due to an increased transport of the transcription factor Pdx1 into the nucleus and an elevated transcription factor activity after CK2 inhibition [18,19,20]. Moreover, the stability of Pdx1 is enhanced, presumably, by disturbing the interaction with PCIF, a ubiquitin ligase adapter protein [18]. The transcription factor USF1 is also a substrate for CK2, and inhibition of CK2 leads to an elevated transcription of the gene encoding insulin [21,22]. The level of intracellular concentration of Ca^2+^ is critical for the synthesis and secretion of insulin from pancreatic β-cells. Two Ca^2+^ channels, Cav2.1 and TRMPM3, are substrates for CK2 and inhibition of CK2 leads to an elevated intracellular Ca^2+^ concentration [23,24]. For these studies, we have used tetrabromobenzotriazol (TBB) [25,26], quinalizarin [27,28], or CX-4945 [29,30] as inhibitors. As TBB was shown to inhibit protein kinases other than CK2, we switched to CX-4945, which was used as a CK2 specific inhibitor to treat various cancers mostly in combination with other drugs [31,32]. Recently however, CX-4945 was identified as a splicing regulator [33] and a selective inhibitor of cdc2-like kinases [34]. Furthermore, about 10 kinases other than CK2 were shown to also be inhibited at low nanomolar concentrations. Thus, according to these results, off-target effects are most likely. Recently, Wells and co-workers published a new compound named SGC-CK2-1, which exhibited a remarkable selectivity for CK2 [35]. Furthermore, proliferation of cells was inhibited in just one analyzed cell line. On the other hand, protein kinase activity of CK2 was strongly inhibited as shown by an impaired Akt S129 phosphorylation. As disruption of the Akt signaling has also been reported for other CK2 inhibitors, these results demonstrated an influence on the down-stream signaling of CK2. As SGC-CK2-1 inhibited CK2 kinase activity ten times more than CX-4945, but seems to have no influence on cell proliferation, we decided to treat pancreatic β-cells with SGC-CK2-1 and, for comparison, with CX-4945. We analyzed the effect on insulin production and secretion from these cells in order to exclude off-target effects more efficiently. We found that both inhibitors had only marginal effects on cell proliferation and cell viability. Both efficiently inhibited CK2 kinase activity, which was determined by Akt Ser129 phosphorylation as well as by phosphorylation of a synthetic CK2 substrate peptide. However, inhibition of CK2 by both inhibitors led to the stimulation of insulin production and secretion. Thus, SGC-CK2-1 turned out to be an appropriate inhibitor to interrogate the biological significance of CK2 for pancreatic β-cells. Due to the high efficiency and specificity of SGC-CK2-1 and the limited influence on cell proliferation, this new inhibitor seems to be highly qualified for further use as potential therapeutic drug in diabetes therapy.

## 2. Materials and Methods

### 2.1. Cell Culture and Treatment

The mouse cell line MIN6 [36,37] was maintained in Dulbecco’s modified Eagle’s medium DMEM supplemented with 10% (*v*/*v*) fetal bovine serum in a humidified atmosphere with 5% CO_2_ at 37 °C. Cells were passaged at a split ratio of 1:3.

The CK2 inhibitors SGC-CK2-1 (Sigma-Aldrich, Taufkirchen, Germany) and CX-4945 (SelleckChem, Munich, Germany) were dissolved in dimethyl sulfoxide (DMSO). A 10 mM stock solution was prepared to treat the cells with the corresponding final concentration. In any case, smaller volumes of stock solution were filled up with pure DMSO to have equal volumes of the solvent in the cell culture dishes. In control experiments, we used an equal volume of the solvent DMSO alone in order to exclude any solvent effects. The final concentration of DMSO in the medium did not exceed 1% (*v*/*v*).

### 2.2. Extraction of Cells and Western Blot Analysis

Cells were harvested by scraping off the plate with a rubber spatula. They were centrifuged (7 min, 4 °C, 400× *g*) and subsequently washed with cold phosphate-buffered saline (PBS). Cells were lysed for 30 min at 4 °C with lysis buffer (10 mM Tris-HCl, pH 7.5, 10 mM NaCl, 0.1 mM EDTA, 0.5% (*v*/*v*) Triton X-100, 0.02% NaN_3_ (*w*/*v*) supplemented with 0.5 mM phenylmethylsulfonyl fluoride (PMSF) and a protease and phosphatase inhibitor cocktail (1:75 *v*/*v*, Sigma-Aldrich, Taufkirchen, Germany). After lysis, cell debris was removed by centrifugation. The protein content was determined using the BioRad protein assay dye reagent (BioRad, Munich, Germany). SDS polyacrylamide gel electrophoresis and Western Blot analysis was performed essentially as described [38]. Equal amounts of protein extracts were loaded onto 12.5% polyacrylamide gels (5 µg for the detection of proinsulin, 15 µg for the detection of CK2 subunits and 25 µg for the detection of total and phosphorylated Akt). Electrophoresis was performed with a Tris based running buffer (25 mM Tris-HCl, pH 8.3, 190 mM glycine, 0.1% sodium dodecyl sulfate) for 1 h at 100 V. After electrophoresis, proteins were blotted onto PVDF membranes by a semi-dry blot procedure using 48 mM Tris, pH 9.2, 39 mM glycine, 20% (*v*/*v*) methanol as blot buffer in a BioRad Trans-Blot-Turbo transfer system (BioRad, Munich Germany) for 7 min at 25 V and 1.3 A. After blotting, the membranes were incubated with the primary antibodies (anti-proinsulin (abcam, Berlin, Germany, ab181542), a polyclonal serum against CK2α, anti-CK2β (Santa Cruz Biotechnologies, Heidelberg, Germany, sc-46666), or anti-α-tubulin (clone DM1A, Sigma-Aldrich, Taufkirchen, Germany)) in TBS (20 mM Tris-HCl, pH 7.5, 150 mM NaCl) supplemented with 0.1% Tween20 (TBS-T) and 1% BSA overnight at 4 °C. Subsequently, membranes were washed twice with TBS-T, and then incubated with the horseradish peroxidase (HRP)-conjugated secondary antibodies (anti-rabbit (HAF-008)) and anti-mouse antibody (HAF-007) from R&D Systems, Abingdon, UK) for 1 h at room temperature. After two further washing steps, the expression of the corresponding proteins was visualized by enhanced chemoluminescence.

### 2.3. In Vitro Phosphorylation

For the determination of the protein kinase activity of CK2 in extracts of treated or untreated cells, we used the synthetic CK2 specific substrate peptide with the sequence RRRDDDSDDD [39]. The enzymatic reaction was performed using radio-labelled [^32^P]γ ATP in an appropriate kinase buffer (50 mM Tris/HCl, pH 7.5, 100 mM NaCl, 10 mM MgCl_2_, 1 mM dithiotreitol (DTT)) containing 20 µg protein/20 µL, which was mixed with 30 µL CK2 mix (25 mM Tris/HCl, pH 8.5, 150 mM NaCl, 5 mM MgCl_2_, 1 mM DTT, 50 μM ATP, 0.19 mM substrate peptide) containing 10 μCi/500 μL [^32^P]γ ATP (Hartmann Analytic, Braunschweig, Germany). The mixture was spotted onto a P81 ion exchange paper. After washing three times with 85 mM H_3_PO_4_ and once with ethanol, the paper was dried and the Čerenkov-radiation was determined in a scintillation counter.

### 2.4. Detection of Insulin Secreted from MIN6 Cells

The determination of secreted insulin was essentially performed as described by Kelly et al. [40]. MIN6 cells were seeded at 3 × 10^4^ cells/well in a 24-well plate. After 24 h, cells were treated with vehicle, SGC-CK2-1 or CX-4945 for 24 h. The medium was removed and after washing with Krebs Ringer buffer KRB (115 mM NaCl, 4.7 mM KCl, 1.28 mM CaCl_2_, 1.2 mM MgSO_4_, 0.1% BSA), the cells were incubated with KRB supplemented with 25 mM glucose for 1 h to induce insulin secretion. After this treatment, insulin was determined in the collected supernatants with the insulin ELISA kit from Invitrogen according to the recommendation of the provider.

### 2.5. RNA Extraction

Cells were harvested by trypsinizing and were subsequently spun down by centrifugation (7 min, 4 °C, 400× *g*). RNA was extracted from treated and control cells using the QIAzol lysis reagent (Qiagen, Hilden, Germany) according to the manufacturer’s instructions. For qRT-PCR, RNA was subsequently washed with 75% ethanol. After drying, RNA was resuspended in RNAse-free water and the concentration was determined using a NanoDrop UV-Vis Spectrophotometer. mRNA was reverse transcribed to cDNA with a qScriber cDNA Synthesis Kit (HighQu, Kraichtal, Germany) according to the manufacturer’s protocol.

### 2.6. Quantitative Real-Time PCR (qRT-PCR)

For amplification of the proinsulin cDNA, we applied the ORA SEE qPCR Green ROX L Mix Kit (HighQu). We strictly followed the recommendations of the manual of the producer. Briefly, 100 ng of total RNA per reaction were reverse transcribed and analyzed in a one-step reaction using the primer combinations shown in Table 1. GAPDH served as an endogenous control for mRNA detection.

### 2.7. Viability Assays

A WST-1 (water-soluble tetrazolium salt-1) assay (cell proliferation reagent Cellpro-Ro from Roche, Rotkreuz, Switzerland) was used to determine the viability of MIN6 cells according to the protocol described in [41]. In brief, 10 µL of WST-1 solution was added to each well of a 96 well plate and mixed with the culture medium. After 30 min the absorbance of the formed dye was determined in a plate reader at 450 nm.

MIN6 cells were seeded in a 24-well plate and cultivated for 24 h. Cells were treated with vehicle, SGC-CK2-1, or CX-4945 (1 µM or 10 µM) for 24 h. The adherent cells were detached with Cell Dissociation Buffer (GIBCO by Fisher Scientific GmbH, Schwerte, Germany), centrifuged, and resuspended with PBS. For the determination of the viability, 10 µL of the cell suspension was stained with a trypan blue solution (0.4%) and counted by a LUNA^TM^ Automated Cell Counter (logos Biosystems, Villeneuve D’Ascq, France) according to the manufacturer’s protocol.

### 2.8. Statistical Analysis

After testing the data of the in vitro experiments for normal distribution and equal variance, differences between the groups were assessed by the one-way analysis of variance (One-way ANOVA). This was followed by the Tukey post hoc test by means of Prism software 8 (GraphPad). Results were expressed as mean ± standard deviation (SD) of at least three or four independent experiments. Statistical significance was accepted as *p* < 0.05.

## 3. Results

### 3.1. Impact of SGC-CK2-1 on Cell Viability

In recent years, different CK2 inhibitors were used as potential tumor therapeutics as they more or less compromised the growth of many tumor cells. The recently published highly specific CK2 inhibitor SGC-CK2-1, however, did not elicit an antiproliferative activity towards most of the tested 140 tumor cell lines, although it inhibited intracellular CK2 activity with a high efficiency [35]. Due to these results, we asked whether the published effects on functions of pancreatic β-cells after inhibition with different CK2 inhibitors are also true for the treatment with SGC-CK2-1.

**Figure 1:**
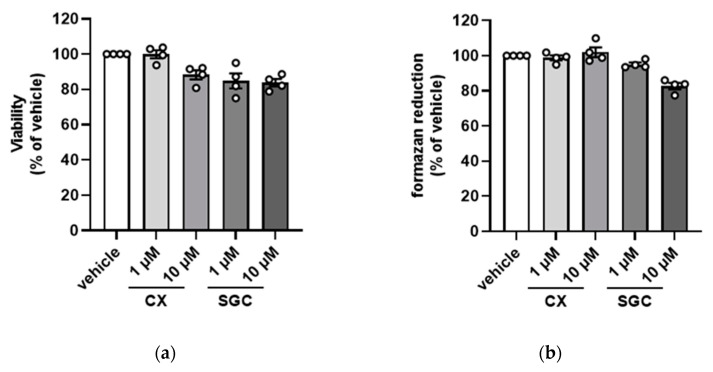
Impact of the treatment of MIN6 cells with SGC-CK2-1 or CX-4945 on cell viability. MIN6 cells were treated with 1 or 10 µM SGC-CK2-1 (SGC) or 1 or 10 µM CX-4945 (CX) for 24 h. (**a**) Viability of cells was determined by counting living cells after trypan blue exclusion. Living cells in the control culture (vehicle) were set 100% and living cells in the treated culture were calculated in reference to it. (**b**) After 24 h treatment, the metabolic activity of MIN6 cells was analyzed by a WST-1 viability assay. Experiments were undertaken at least in quadruplicate and statistical analysis was performed as described in “Material and Methods”.

For our experiments we used the mouse insulinoma cell line MIN6 [36,37], which has morphological characteristics of pancreatic β-cells and shows glucose-inducible insulin secretion comparable to cultured normal mouse islet cells. There are numerous reports showing that inhibition of CK2 may have an impact on the viability of cells (for review see: [42,43,44,45]). Therefore, we treated MIN6 cells with the recently published inhibitor SGC-CK2-1 (1 and 10 µM) or with the well-established inhibitor CX-4945 (1 and 10 µM). After 24 h, we performed a dye exclusion test using trypan blue and determined the ratio of viable cells. As shown in Figure 1a, we observed essentially no reduction in viability after treatment with 1 µM CX-4945 compared to the DMSO control. Upon application of 10 µM CX-4959, the viability was reduced to 90%. Treatment with 1 µM SGC-CK2-1 caused a drop of viability to 85% which was no further exceeded by the higher concentration.

Viability of cells can also be tested by analyzing the metabolic activity of dehydrogenases. Cells were treated with the inhibitors as described for the dye exclusion test; the viability of the cells was determined in a WST-1 assay. The results shown in Figure 1b confirmed the data of the previous experiment. Treatment with CX-4945 had no influence on the viability of cells, whereas treatment with SGC-CK2-1 led to a reduction in viability to about 85% when using 10 µM SGC-CK2-1. Thus, with both assays, we found no influence of CX-4945 and a weak influence of SGC-CK2-1 on the viability of MIN6 cells.

### 3.2. Impact of SGC-CK2-1 on Endogenous CK2 Activity

**Figure 2:**
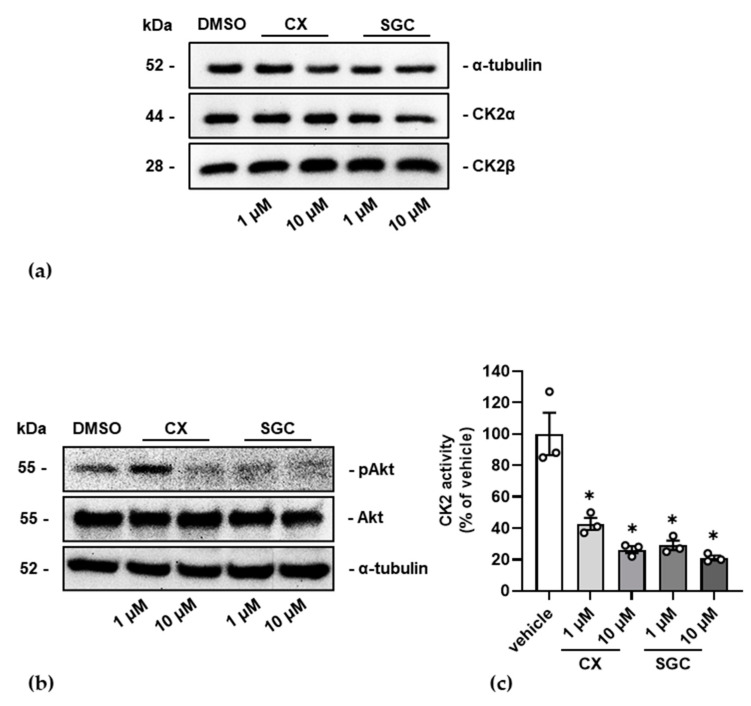
Influence of CK2 inhibitors SGC-CK2-1 and CX-4945 on CK2 expression and CK2 kinase activity. MIN6 cells were treated with 1 or 10 µM CX-4945 (CX) or 1 or 10 µM SGC-CK2-1 (SGC) for 24 h. Control cells were incubated with an equal volume of the vehicle DMSO. Proteins were extracted and equal amounts were either loaded on a 12.5% SDS polyacrylamide gel and blotted onto a PVDF membrane or subjected to an in vitro phosphorylation assay. (**a**) Representative immunoblot analysis of MIN6 cell extracts for the detection of the catalytic subunit CK2α and the non-catalytic CK2β subunit; the detection of α-tubulin served as loading control. (**b**) Representative immunoblot analysis for the detection of total Akt and Akt phosphorylated at the CK2 site serine 129 (pAkt). α-tubulin served as loading control. (**c**) Cell extracts were incubated with [^32^Pγ]ATP and the synthetic CK2 substrate peptide RRRDDDSDDD. After the kinase reaction, labelled phosphate incorporated into the peptide was determined by Čerenkov counting. Activity measured in control extracts was set 100% and the activity of treated extracts calculated in reference to it. Statistical analysis was performed as described in “Material and Methods”. * Statistical significance was accepted with a *p*-value of at least *p* < 0.05.

In the next step, we asked whether treatment of MIN6 cells with SGC-CK2-1 would influence the expression of CK2α and CK2β. Cells were treated with 1 or 10 µM SGC-CK2-1 and, for comparison, with the same concentration of CX-4945. After 24 h cells were harvested and lysed. Total cellular proteins were analyzed by SDS gel electrophoresis followed by Western blot analysis with CK2α or CK2β specific antibodies. As shown in Figure 2a, neither SGC-CK2-1 nor CX-4945 had an influence on the expression of CK2α or CK2β. Next, we asked whether the inhibitors also inhibit the intracellular CK2 activity. We treated the cells as described above and subsequently analyzed the expression of the endogenous CK2 substrate Akt and the phosphorylation of Akt S129 with a phospho-specific antibody. Furthermore, we also analyzed the ability of CK2 to phosphorylate the synthetic CK2-specific peptide substrate RRRDDDSDDD [46].

The Western blot analysis of the known CK2 phosphorylation site S129 in Akt (pAkt) compared with the amount of total Akt demonstrated the inhibiting impact of SGC-CK2-1 or CX-4945 on intracellular CK2 activity (Figure 2b). By using CX-4945, phosphorylation at Akt S129 was efficiently reduced with a 10 µM concentration. Treating the cells with 1 µM SGC-CK2-1 prevented the Akt S129 phosphorylation already nearly completely.

To verify the inhibitory activity with another substrate, we used the synthetic CK2 substrate peptide RRRDDDSDDD. We performed an in vitro phosphorylation assay with extracts of SGC-CK2-1 or CX-4945 treated cells and radiolabeled ATP as phosphate donor. The phosphate incorporation into the peptide was determined by Čerenkov counting. Activity in extracts of DMSO treated control cells was set 100% and residual activity in extracts of SGC-CK2-1 or CX-4945 treated cells was referred to it. As shown in Figure 2c CK2 activity was efficiently reduced with both inhibitors to a level of roughly 20% residual activity using 10 µM CX-4945 or both concentrations of SGC-CK2-1.

Thus, both CK2 inhibitors efficiently inhibited endogenous CK2 without exerting any influence on the expression of the protein and without significantly impairing the viability and proliferation of cells.

### 3.3. Impact of SGC-CK2-1 on Insulin Production and Secretion

In recent years, we intensively studied the importance of protein kinase CK2 for functions of pancreatic β-cells. The most obvious effects were an enhancement of insulin transcription—presumably by stabilization of PDX-1—and insulin secretion after inhibiting protein kinase CK2 by different CK2 inhibitors. We now wanted to know whether SGC-CK2-1 might also induce insulin production and secretion.

**Figure 3:**
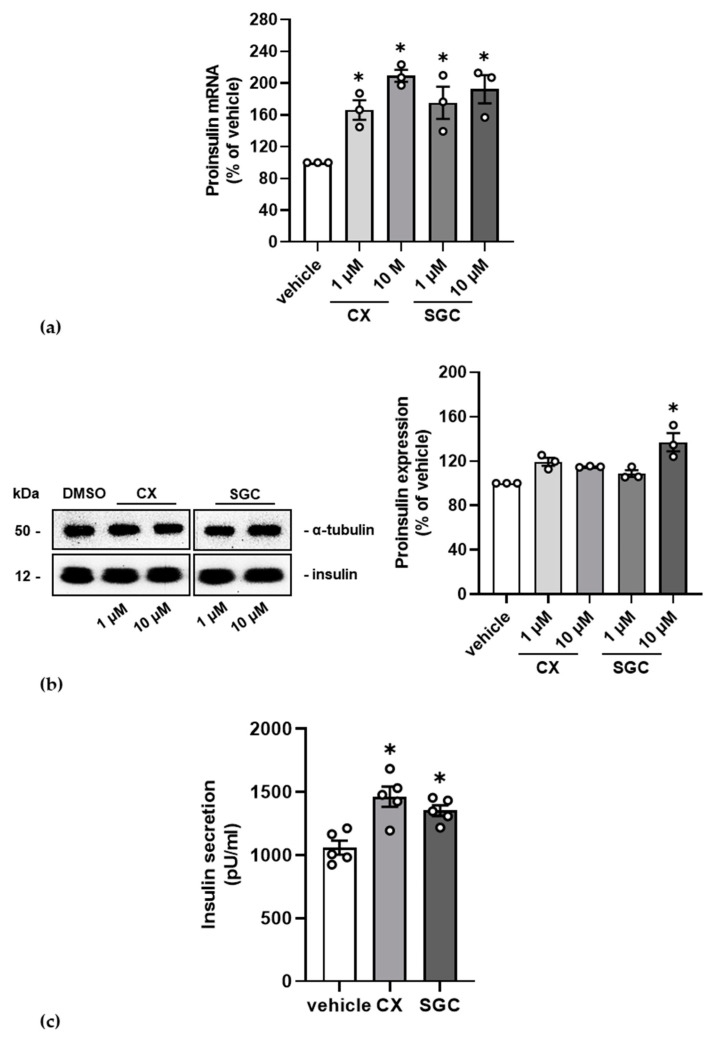
Influence of CK2 inhibition on insulin expression and secretion. MIN6 cells were treated with 1 or 10 µM SGC-CK2-1 (SGC) or 1 or 10 µM CX-4945 (CX) for 24 h. Control cells were incubated with an equal volume of the vehicle DMSO. (**a**) Cells were harvested, and total RNA was isolated using the QIAzol lysis reagent. The mRNA amount of insulin was determined using qRT-PCR. After normalization to GAPDH, the amount of insulin mRNA of control-treated cells was set 100% and the amount of treated mRNA cells calculated in reference to it. (**b**) Cells were harvested, proteins were extracted, and equal amounts were loaded on a 12.5% SDS polyacrylamide gel and blotted onto a PVDF membrane. A representative immunoblot analysis of MIN6 cell extracts for the detection of proinsulin is shown; the detection of α-tubulin served as loading control. Signals for proinsulin from three independent experiments were analyzed by a densitometric scan and normalized to the arbitrary amount of the loading control α-tubulin. The relative mean amount of proinsulin +/− SD for the different treatments is shown as bar graphs. (**c**) After a glucose stimulus, secreted insulin was determined in the cell culture supernatant with the insulin ELISA kit. Statistical analysis in all experiments was performed as described in “Material and Methods”. * Statistical significance was accepted with a *p*-value of at least *p* < 0.05.

In the first instance, we checked the effect of CK2 inhibition on insulin transcription by qRT-PCR. MIN6 were treated with 1 and 10 µM SGC-CK2-1 and, for comparison, also with 1 and 10 µM CX-4945 or with the solvent DMSO for control. mRNA was isolated, reverse transcribed and subjected to a qPCR analysis with insulin specific primers. The message was normalized to the housekeeping gene GAPDH. The bar graphs in Figure 3a show that upon addition of both CK2 inhibitors the message for insulin increased up to two-fold compared to the DMSO control.

We next asked whether the increase in insulin transcription was also mirrored in protein expression. We treated the cells as described above and analyzed the expression of the precursor form proinsulin in a Western blot analysis and presented the relative amount after normalization to the loading control α-tubulin (Figure 3b). We repeatedly found an elevated level of proinsulin protein in treated cells. The increase was, however, lower than expected from the qRT-PCR analysis.

To cope with glucose concentrations about the physiological level of 5 mM, it is important that insulin is secreted from the β-cell to induce processes which lower high glucose amounts after feeding. As we observed in former experiments an enhanced secretion of insulin after inhibition of CK2, we now asked whether the treatment of MIN6 with SGC-CK2-1 might lead to the same result. Again, we treated the cells with SGC-CK2-1 and, for comparison, also with CX-4945, and determined the secreted insulin. With both inhibitors, we observed a significant increase in insulin secretion by 1.5-fold. Thus, we could demonstrate that by inhibiting endogenous protein kinase CK2 in pancreatic β-cells with the recently published inhibitor SGC-CK2-1 led to the same effects on β-cell specific functions than with the long established CK2 inhibitor CX-4945.

## 4. Discussion

The observation that CK2 expression and CK2 protein kinase activity is elevated in rapidly proliferating cells has stimulated the search for inhibitors of the kinase activity with the goal to use inhibitors as anti-cancer agents. Modifying existing chemical structures and substitutions led to an increase in the specificity of these inhibitors. By using larger panels of different kinases as well as the use of the inhibitors in different biological systems, it turned out that at least the class of ATP competitive CK2 inhibitors have off target effects [29,33,35,47,48]. These observations did not considerably influence the use of these inhibitors, often in combination with other drugs, for the treatment of cancer, where growth arrest and eventually apoptosis was intended [12,49]. Beside cancer cells, we have used several CK2 inhibitors in pancreatic β-cells where we could show an elevated production and subsequent secretion of insulin after CK2 inhibition [17,22,23]. Additionally, in pancreatic β-cells we have faced the problem with possible off-target effects of the used inhibitors. Recently, a new CK2 inhibitor, namely SGC-CK2-1, was published [31], which had no anti- proliferative activity against 140 different cancer cell lines. Wells et al. [35] further found that SGC-CK2-1 inhibited CK2 kinase activity more efficiently than the commonly used CX-4945 inhibitor at the same concentrations. Recently, Licciardello and Workman [50] encouraged the scientific community to embrace SGC-CK2-1 as the new gold standard to interrogate the biological significance of CK2. The observations described by Wells et al. [35], together with the paper by Licciardello and Workman, stimulated us to compare both SGC-CK2-1 and CX-4945 in pancreatic β-cells with regard to their influence on cell viability, kinase activity, protein expression and production, and secretion of insulin. It turned out that both inhibitors have only a minor effect on cell viability, with a reduction of around 10–15%, compared to the untreated cells. There might be a slightly greater reduction for SCG-CK2-1 treated cells than for CX-4945 treated cells. The results obtained here for CX-4945 treated MIN6 cells are in agreement with earlier observations with non-cancer ARPE-19 cells, where CX-4945 efficiently inhibited CK2 kinase activity without an influence on cell viability [51].

CK2 kinase activity is often determined by measuring the phosphorylation of one of the down-stream signaling molecules, namely Akt, which is phosphorylated by CK2 at serine129 [52]. Phosphorylation of Akt is detected by a phospho-specific antibody directed against the CK2 phosphorylation site serine 129. As shown here, Akt 129 phosphorylation is inhibited by both inhibitors where SCG-CK2-1 is effective already at 1 μM, whereas CX-4945 strongly inhibits Akt 129 phosphorylation at a concentration of 10 μM. A very similar result was obtained when a peptide with the CK2 consensus sequence RRRDDDSDDD [39] was used as substrate. Both inhibitors do not have an influence on the expression of CK2α and CK2β as shown by a Western blot analysis. Thus, the reduction in the CK2 kinase activity is not due to reduction in the level of CK2 proteins. We have shown that inhibition of CK2 kinase activity leads to an elevated level of the transcription factor Pdx-1 in the nucleus, which acts there as a transcription factor increasing the expression of insulin [20]. As shown here, both inhibitors stimulated the expression of proinsulin, which was detected by qRT-PCR and Western blots. We observed that the mRNA expression for insulin was considerably enhanced after CK2 inhibition by both inhibitors. The proinsulin protein expression was only slightly increased in the presence of the inhibitors; SGC-CK2-1 seems to be more efficient than CX-4945 when a 10 μM concentration was used. We have previously shown that not only the synthesis of insulin was enhanced after CK2 inhibition, but also the secretion of insulin [23]. Here, we have shown that both inhibitors forced the secretion of insulin from pancreatic β-cells as measured by an insulin ELISA. This result might also explain that the level of proinsulin is only slightly elevated after CK2 inhibition as the secretion is elevated simultaneously. In summary, our results strongly suggest that the elevated production and secretion of insulin from pancreatic β-cells is not due to off-target effects, but specific for the inhibition of the CK2 kinase activity. These results suggest SGC-CK2-1 as a new tool to target CK2 in pancreatic β-cells in order to improve the treatment of diabetes.

## Figures and Tables

**Table 1:** Primer pairs applied in qRT-PCR.

Target	Direction	Sequence
Proinsulin	forward	5′-GGG GAG CGT GGC TTC TTC TA-3′
Proinsulin	reverse	5′-GGG GAC AGA ATT CAG TGG CA-3′
GAPDH	forward	5′-CGG TGC TGA GTA TGT C-3′
GAPDH	reverse	5′-TTT GGC TCC ACC CTT C-3′
